# Is removal of the internal fixation after successful intervertebral fusion necessary? A case–control study based on patient-reported quality of life

**DOI:** 10.1186/s13018-022-03031-6

**Published:** 2022-03-04

**Authors:** Shangbo Niu, Dehong Yang, Yangyang Ma, Shengliang Lin, Xuhao Xu

**Affiliations:** Department of Orthopeadic Spine, Nanfang Hospital, Southern Medical University, 1838 Guangzhou North Ave, Guangzhou, 510515 China

**Keywords:** Internal fixation, Removal, Lumbar degenerative disease, SF-36

## Abstract

**Background:**

Intervertebral fusion and internal fixation are often applied to patients with lumbar spinal disease. Whether to remove the internal fixation after successful fusion remains uncertain, but such a question needs to be explored in light of concerns regarding patients’ quality of life and health insurance. We sought to probe if the removal of internal fixation after successful lumbar intervertebral fusion affects patients’ quality of life.

**Methods:**

This was a real-world retrospective case–control study. Data of 102 patients who had undergone posterior lumbar fusion with cage and internal fixation to treat lumbar degenerative diseases were extracted from a single center from 2012 to 2020. Fifty-one patients had undergone internal fixation removal surgery, and 51 controls who retained internal fixations were matched according to demographic and medical characteristics. The quality of life of patients based on the Medical Outcomes Study Short Form 36 (SF-36) scale and their self-assessment were surveyed.

**Results:**

There was no statistical difference in the overall score of the SF-36 questionnaire between the two groups, but the general health (GH) subscore was lower in the case group than in the control group (*P* = 0.0284). Among those patients who underwent internal fixation removal, the quality of life was improved after instrument removal as indicated by an increased overall score (*P* = 0.0040), physical functioning (PF) (*P* = 0.0045), and bodily pain (BP) (*P* = 0.0008). Among patients with pre-surgery discomfort, instrument removal generated better outcomes in 25% and poor outcomes in 4.2%. Among patients without pre-surgery discomfort, instrument removal generated better outcomes in 7.4% and poor outcomes in 11.1%.

**Conclusion:**

Among patients who achieved successful posterior lumbar internal fixation, whether or not to remove the fixation instruments should be evaluated carefully. In patients experiencing discomfort, instrument removal could improve their quality of life, but the benefits and risks should be comprehensively explained to these patients. Instrument removal should not be routinely performed due to its limited or even negative effect in patients who do not report discomfort before surgery.

## Background

The strategy of posterior discectomy followed by lumbar fusion and internal fixation is commonly adopted to treat patients with lumbar degenerative disease (LDD) [1]. In the United States, there are more than 300,000 lumbar fusion operations performed every year [2]. With the growing senior population, the incidence of LDD and lumbar fusion operations is expected to persist at a high level [3]. Internal fixation is essential for successful fusion by providing the spine stability that allows for bone growth and union. However, after successful intervertebral fusion, solid stability would be achieved; thus, internal fixation becomes redundant and unnecessary, and in this context, theoretically, the internal fixation instruments could be removed without any side effects. Patients often ask whether it is necessary to conduct an operation for removing these instruments [4].

Internal fixation removal is a common treatment option in patients with healed fractures, either in the extremities or the lumbar spinal column. In Finland, 81% of patients with a variety of fracture types underwent implant removal operations after bone healing [5]. Internal fixation extraction accounts for almost one-third of all selective orthopedic operations [6]. In patients with healed spinal fractures, internal fixation removal is routinely conducted to restore the spine’s mobility and reduce the possibility of adjacent segment degeneration or fractures, although such a strategy has been challenged from different angles by some investigators such as self-based assessment of patients and no significant difference in radiology or function [7, 8].

In practice, whether internal fixations located at intervertebral fused segments should be removed has not garnered a consensus among clinicians [9, 10] due to a lack of evidence-based clinical investigations. In the present study, we proposed that the removal of internal fixations (transpedicular screws and rods, which are inserted into the vertebral pedicle to provide fixation) after successful intervertebral fusion would not affect patients’ quality of life. A case–control study was designed to compare the quality of life of patients who underwent instrument removal with those who did not in order to evaluate the impact of internal fixation removal on patients’ quality of life.

## Methods

### Study design and patients

This was a retrospective case–control study. Patients in the case group who underwent an operation for internal fixation removal at Nanfang Hospital of Southern Medical University from 2012 to 2020 were pooled. These patients had clear postoperative low back pain that was difficult to diagnosed by laboratory report and Imaging results. Those who were excluded from this study were as follows: (1) patients who received internal fixation due to spinal fracture; (2) patients who underwent fixation removal or replacement due to failure of internal fixation and fusion, surgical site infection, or another unplanned second operation due to adverse events associated with fixation surgery; (3) patients with whom contact could not be maintained; and (4) patients with severe diseases or postoperative complications that affected their quality of life.

**Fig. 1:**
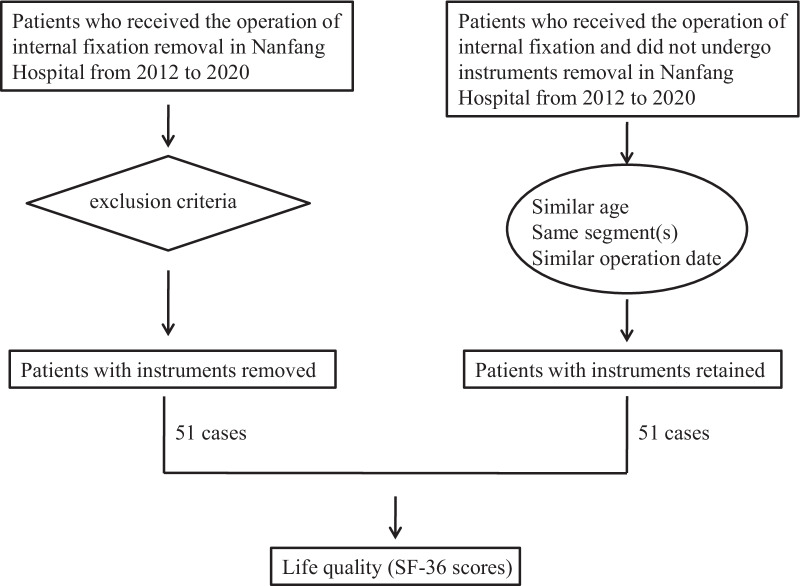
Flow-chart of choosing cases

For each case, a control was matched according to the following criteria: (1) internal fixation was retained, (2) same sex, (3) similar age (± 5 years), (4) the original operations of intervertebral fusion and internal fixation were performed at the same lumbar segment, (5) the date of the original operation was within three months of study participation (if more than one patient qualified to be a matched control, the one with the closest operation time point was chosen as the control), and (6) there were no severe diseases or postoperative complications affecting the quality of life (Fig. 1).

### Surgical technique

The surgical techniques of posterior lumbar interbody fusion (PLIF) or transforaminal lumbar interbody fusion (TLIF) were performed under general anesthesia. First, a posterior longitudinal middle incision was made; then, after the erector spinae and multifidus muscles were dissected from the spinous process and lamina, the ipsilateral lamina and/or part of facet joints were resected. After protecting nerve roots, the annulus fibrosis was cut through, and the disc was removed; then, bone graft bed preparation on an endplate was completed. After discectomy, a standard intervertebral fusion with autograft bones (isolated from resected bone tissue during decompression) and bilateral transpedicular screws were placed [11].

All patients included in this study had similar open procedures. The instruments removal surgery was performed under general anesthesia. A 4–6 cm posterior midline longitudinal incision was made along the previous surgical incision, the skin and subcutaneous tissues were incised layer by layer. Then the tissue above the lumbodorsal fascia was dissected till 2 cm from midline. The screws could be touched and then a small incision of lumbodorsal fascia (3 cm in length on each side) right above the screws was made. The muscles were bluntly separated and the connective tissue around the instrumentation was cleared. The screws and rods were removed by the tools that originally used in instrumentation. The drainage was placed and the deep fascia, hypodermis and derma were sewed up layer by layer. The drainage was removed the next day and patients were discharged in 3 days.

### Measurement

Demographic data, such as gender and age, medical information such as operation time, bleeding, postoperative drainage, hospital stay and inpatient total cost, and data regarding operation time were collected from the electronic inpatient system.

Follow-up assessments were carried out at the outpatient section of Nanfang Hospital or over phone. Before entering the follow-up period, patients were informed about this study, and their consent for inclusion was recorded, and either the patients themselves or their close family members ensured the completion of follow-up. The Medical Outcomes Study Short Form 36 (SF-36) scale was used to measure the outcome [12] and includes the following eight sections: physical functioning (PF), physical role functioning (RP), bodily pain (BP), general health (GH), vitality (VT), social functioning (SF), emotional role functioning (RE), and mental health (MH). Total SF-36 scores were calculated by assuming that each section has the same weight coefficient. During the first follow-up visit, the SF-36 quality-of-life scale items were described one by one, and the answer to each was not recorded without the patient having confidence in their understanding.

Patients’ overall perceptions about the results of the instrument removal surgery were also recorded. The outcome was classified as either better, similar, or worse compared with the pre-surgery condition.

### Statistical analysis

Categorical variables, such as the number of different types of patients, are presented using frequencies and percentages, while continuous variables are presented using mean and standard deviation values, as shown in Table 1. The paired *t*-test (Mann–Whitney U test) was used for analysis purposes, and *P* < 0.05 was used to indicate a statistical difference between the two groups. Statistical analysis was performed using GraphPad Prism version 8 (GraphPad Software Inc., La Jolla, CA, USA). All missing data were replaced by average values.

**Table 1:** Demographic characters of the patients

	Instruments removed	Instruments retained	*P* value
Case number	51	51	
Age (year)	40.16 ± 10.90	40.31 ± 10.93	0.7689
Gender (M:F)	35:16	35:16	
Single segment	42	42	
Double segments	9	9	
Operation time (min)	174.33 ± 44.18	178.12 ± 63.29	0.7322
Bleeding (ml)	205.43 ± 87.61	201.11 ± 108.09	0.8224
Drainage (ml)	193.70 ± 138.64	231.11 ± 120.73	0.1280
Hospital stay (day)	10.67 ± 4.35	11.16 ± 3.98	0.5837

## Results

Data of 51 patients were obtained from the medical records of Nanfang Hospital and that of corresponding controls with similar age and sex and the same fused segment was also extracted. As for surgical details about the fusion operation, there was no statistically significant difference in the operation time, bleeding volume, drainage volume, or hospital stay length (Table 1).

Total SF-36 scores did not exhibit a statistically significant difference between the case and control groups (Table 2). Even in the module of GH, the cases scored significantly lower than the controls. When comparing the pre- and post-surgery scores in the case group (Table 3), the total score and subscores of the PF, RP, and BP modules were significantly improved following instrument removal surgery (*P* < 0.05).

**Table 2:** SF-36 scores reported by the patients who underwent instruments removed and those with instruments retained

SF-36	Case	Control	*P* value
Total	85.10 ± 9.27	86.17 ± 14.06	0.6559
PF	92.02 ± 8.02	89.57 ± 13.34	0.3205
RP	74.49 ± 28.94	80.43 ± 30.79	0.3649
BP	84.82 ± 14.75	83.07 ± 19.15	0.6531
GH	74.96 ± 13.38	82.74 ± 19.08	0.0284*
VT	82.00 ± 9.33	83.87 ± 14.25	0.4360
SF	93.43 ± 12.02	94.04 ± 14.00	0.8144
RE	95.14 ± 11.78	87.63 ± 28.61	0.0657
MH	86.86 ± 7.80	88.04 ± 12.14	0.5055

*Significant difference

**Table 3:** SF-36 scores reported by the patients before and after instruments removal

SF-36 scores	Before	After	*P* value
Total	82.96 ± 9.68	85.10 ± 9.27	0.0040*
PF	88.95 ± 10.16	92.02 ± 8.02	0.0045*
RP	71.41 ± 31.93	74.49 ± 28.94	0.0600
BP	79.22 ± 14.94	84.82 ± 14.75	0.0008*
GH	72.77 ± 13.90	74.96 ± 13.38	0.1508
VT	83.03 ± 8.17	82.00 ± 9.33	0.1005
SF	91.95 ± 13.55	93.43 ± 12.02	0.1491
RE	92.31 ± 15.05	95.14 ± 11.78	0.0815
MH	86.03 ± 7.43	86.86 ± 7.80	0.2104

*Significant difference

The 51 patients in the case group were subdivided into two subgroups according to whether the patients reported preoperative discomfort during interviews. In 24 patients with back and/or leg pain, six (25%), 17 (70.8%), and one (4.2%) patient(s), respectively, reported that the operations led to better, similar, and worse outcomes, respectively. In 27 patients who did not have preoperative discomfort, two (7.4%), 22 (81.4%), and three (11.1%) patients reported that the operations led to better, similar, and worse outcomes, respectively (Table 4).

**Table 4:** Surgical outcomes reported by patients experienced instrument removal

Outcome	Back pain (*n* = 18)	Leg pain (*n* = 6)	No discomfort (*n* = 27)
Better (*n* = 8)	4	2	2
Similar (*n* = 39)	13	4	22
Worse (*n* = 4)	1	0	3

The symptoms of four patients who reported worse outcomes varied in each person, and they reported "easy fatigue", "limited heavy work", "numbness in the lower back and legs after sitting and standing for a long time", or "numbness of the original affected leg" respectively.

No complications of delayed skin healing or infections or hematoma occurred in the patients. Patients were subdivided into two groups including 24 patients with interval of surgeries less than 1000 days and 27 patients with interval more than 1000 days. The overall score of SF-36 and surgical information about the second operation was no statistically significant difference (Table 5).

**Table 5:** The overall score of SF-36 and surgical information of removal surgeries between patients whose interval of surgeries less than 1000 days and more than 1000 days

	< 1000 days interval	> 1000 days interval	*P* value
Case number	24	27	
Total score	84.10 ± 8.64	81.48 ± 10.38	0.3489
Operation time (min)	80.88 ± 43.28	71.62 ± 18.46	0.3337
Bleeding (ml)	43.13 ± 27.91	41.19 ± 26.51	0.8065
Drainage (ml)	70.21 ± 58.57	74.39 ± 58.83	0.8063

## Discussion

Lumbar fusion and fixation are widely performed for LDD patients, but whether or not it is necessary to remove the fixation instruments after fusion remains unknown. To investigate, we designed a quality of life–oriented case–control study and collected some interesting information. When comparing the post-removal quality of life of cases against that of controls retaining fixation instruments, no statistically significant difference was found in the overall SF-36 score and almost all individual modules, except GH, the score of which was lower among cases than controls. However, when comparing the pre- and post-surgery quality of life of the cases, a significant improvement was found in the overall score of SF-36 and subscores of the PF, BP, and RP modules. In order to exclude the possibility of the effect of surgical difficulty by comparing the surgical information of patients with different intervals between two operations. Taken together, patients who underwent instrument-removal surgery achieved an improvement in their quality of life, which was contrary to what was expected.

Instrument removal is routinely performed after spinal fracture healing to recover the movable segments and reduce the possibility of adjacent segmental degradation. Axelsson et al. reported that late implant removal could restore mobility in fractured segments without bone grafting, depending upon the outcomes of radiostereometry analysis [13]. However, whether a decision could be made based on a patient’s symptoms rather than the imaging evidence about the necessity of removal surgery is unclear [14]. Oh et al. reported that conducting the removal operation within 12 months after the first surgery could ensure better recovery of the range of motion (ROM) but advised that this surgery should only be recommended to patients with symptoms [15]. Chou et al. reported a series of 69 patients who were followed up for up to 66 months (range: 47–108 months); of these cases, 47 patients underwent implant removal and 22 did not, and there were no statistically significant differences in the radiological and functional outcomes between these two groups [8], even though eight patients in the implant retention group experienced screw breakage. However, Jeon et al. reported that, in a case–control study with 45 patients in each group, at 18.3 ± 17.6 months after fixation removal, the range of segmental motion was increased significantly, and pain (as evaluated with a visual analog scale) and physical function (as evaluated using the Oswestry Disability Index) were improved [14].

Unlike the predictable benefits of segment motion reservation after fracture healing, instrument removal after intervertebral fusion seems to be unnecessary. In our investigation, no statistically significant difference was revealed between the cases that underwent instrument removal and the controls who retained the instruments, which indicated that patients’ quality of life was set before instrument removal; however, this does not mean that instrument removal is unnecessary. This study was not a prospective randomized trial, and mostly, the instrument removal surgeries were performed only upon the request of patients who were experiencing discomfort and a lower quality of life; in other words, the patients in the instrument removal group already had a lower quality of life before the instrument removal surgery than those in the control group; so, instrument removal did not lead to an overall better outcome relative to instrument retention, even though individual patients may have benefited from the operation. Indeed, positive benefits of instrument removal could be summarized based on the fact that statistically significant differences were found between before and after operation in the case group. Our results are also supported by some previously published case reports. Ucler et al. have reported 30 patients who had successfully completed lumbar fusion were selected. A more significant reduction was revealed in VAS and ODI scores at 1st and 6th month postoperatively [9]. Ak et al. reported a series of 25 patients who underwent lumbar fusion and fixation because of vertebral fracture (*n* = 9 cases), instability of multi-laminectomy (*n* = 12 cases), and recurrent lumbar disc herniation (LDH) (*n* = 4 cases). After removing their implants, the mean visual analog scale score was decreased from 8.08 to 3.36 points, but five (20%) patients (including all four recurrent LDH cases) did not report a reduction in pain. Meanwhile, no patient reported complete relief from pain [10] in an investigation of 57 patients who underwent implant removal after fusion and fixation because of pain and discomfort; the initial diagnoses in this study were fracture (40%) and degenerative spine disease (58%). In a survey conducted from 6 to 24 months after surgery, 35 (61%) patients stated that they had experienced some benefits of the operation, but only seven (12%) of them felt preoperative discomfort had been restored completely; moreover, 36 (63%) patients would undergo the operation again [6]. Therefore, instrument removal could improve the quality of life of patients experiencing discomfort.

In our study, the 51 patients who underwent instrument removals were subdivided into two groups, and their overall comments about instrument removal surgery were compared. Among those patients with back and/or leg discomfort, 25% reported that instrument removal led to better results and 4.2% reported that the surgery led to worse results. Overall, most of the patients experiencing discomfort reported that the additional surgery did not alter their condition. To some extent, patients’ comments reflected whether their expectations were met by the surgery; so, the results were slightly different from the quality of life scoring as mentioned above. Nevertheless, the results indicated that instrument removal could improve patients’ quality of life but might not fully achieve their expectations.

In our study, 27 patients requested instrument removal for reasons other than discomfort, and most of them reported that the postoperative outcome was similar to their status prior to surgery; however, three (11.1%) patients reported a worsened status after surgery. As such, patients without discomfort may not be encouraged to remove the fixation. In practice, many clinicians have considered the removal of screws and rods to be unnecessary, even in cases with some indications met (e.g., the breakage of screws or rods) [16]. Further, new fractures still often occur in the area connecting the fused segments and their adjacent non-fused segments after the instrument was removed [17]. As such, routine removal should not be recommended.

Whether or not to remove the fixation after lumbar intervertebral fusion is a commonly considered but important question that needs to be addressed by evidence-based studies because it can influence patients’ quality of life as well as health insurance policies. Our investigation focused on surgeries for LDD in a break away from previous reports that were mainly case series in which the initial diagnosis varied, including LDD, deformities, and fractures [6, 10, 18]. There were also some shortcomings associated with our study design. First, in this retrospective study, patients could not be randomized; so, those who underwent instrument removal had a lower quality of life than their counterparts. Second, the clinical outcome evaluation methods did not include psychogenic measurements, which may have affected the results. Therefore, a randomized clinical trial needs to be conducted in future.

In conclusion, among patients who underwent lumbar intervertebral fusion and fixation, the decision to remove the internal fixation after successful fusion should be thoroughly validated with consideration of the pros and cons. Patients’ quality of life was mainly linked to the fusion and fixation surgery. For patients with discomfort, instrument removal could improve their quality of life; however, instrument removal should not routinely be recommended because, in patients without discomfort, instrument removal did not improve, or even worsened, their quality of life.

## Data Availability

The datasets used and/or analysed during the current study are available from the corresponding author on reasonable request.

## Abbreviations

SF-36: Medical Outcomes Study Short Form 36
LDD: Lumbar degenerative disease
LDH: Lumbar disc herniation
PLIF: Posterior lumbar interbody fusion
TLIF: Transforaminal lumbar interbody fusion
ROM: Range of motion
